# The lightness/pitch crossmodal correspondence modulates reported dominance in binocular rivalry

**DOI:** 10.3758/s13414-026-03320-w

**Published:** 2026-08-12

**Authors:** Mick Zeljko, Rhianna Eather, Bryan Sim, Philip M. Grove, Ada Kritikos

**Affiliations:** School of Psychology, The University of Queensland, St. Lucia, Queensland 4072 Australia

**Keywords:** Multisensory Processing, Binocular vision: Rivalry/Bistable Perception

## Abstract

**Abstract:**

Crossmodal correspondences (CMCs: systematic associations between feature dimensions across sensory modalities) are known to facilitate performance in simple detection and discrimination tasks and can bias reports of ambiguous figures. The present study tested whether a lightness/pitch CMC biases reported dominance in binocular rivalry. Across three experiments, participants viewed dichoptically presented black and white circles accompanied by relatively high- or low-pitched tones. Dominance reports were coded as congruent when they matched the lightness/pitch correspondence (white/high, black/low) and incongruent when they did not (white/low, black/high). In two experiments, participants reported whether the dominant percept was black or white (direct task); in a third, they judged line orientation while lightness was irrelevant (indirect task). Congruency effects were observed only in the direct task, and only at longer timescales: in intermittent presentation, at 5-s and 8-s exposures; and in continuous presentation, for 5-s sound alternations with responses following tone changes by 1.6–3.2 s, peaking at about 2.6 s. No reliable effects were found in the indirect task. Dominance response durations averaged approximately 2.5 s in the direct task but were significantly longer, approximately 3.5 s, in the indirect task. These results suggest that a pre-existing, untrained lightness/pitch correspondence can bias reported rivalry dominance, but only when the relevant feature is attended and during temporal windows aligned with natural rivalry dynamics. Because dominance was measured by report, the findings are best interpreted as effects on reported dominance rather than as definitive evidence for purely perceptual modulation; nevertheless, they are consistent with subtle crossmodal influences during phases of rivalry instability.

**Open practices statement:**

The data for this study are publicly available on the Open Science Framework (OSF) at https://osf.io/u3ycm/overview?view_only=9fc10ddf4d6c4af8a7d70f2e06c2aef6. The study was not preregistered.

## Introduction

Perception is not a direct reflection of the external world but rather the product of neural processes that resolve ambiguous sensory inputs into coherent experiences (Ernst & Bülthoff, 2004; Gilbert & Sigman, 2007; Long & Toppino, 2004; Shams & Beierholm, 2010; Zeljko & Grove, 2021). Multisensory integration is one mechanism that aids this resolution by combining information across modalities to enhance detection and discrimination (Alvarado et al., 2007; Green & Angelaki, 2010; Stein et al., 2020). However, deciding which signals belong together is itself a source of ambiguity. Even when strong drivers of integration like spatial and temporal cues align (Ernst & Bülthoff, 2004; Evans & Treisman, 2010; Meredith & Stein, 1983), multiple interpretations can remain viable (Eramudugolla et al., 2011; Klapetek et al., 2012; Parise et al., 2013; Spence, 2011). For example, resolving which auditory source corresponds to which visual object in a dynamic scene may remain uncertain despite spatiotemporal consistency.

Crossmodal correspondences (CMCs) provide an additional class of cues that can bias such perceptual decisions. CMCs are systematic associations between features across modalities, such as high pitch with lighter, smaller, or higher-positioned visual stimuli, and low pitch with darker, larger, or lower ones (Marks, 1987; McEwan et al., 2024a; Parise & Spence, 2012; Spence, 2011; Zeljko et al., 2019). These correspondences extend beyond low-level features to more complex domains such as taste–shape or music–colour (Ben-Artzi & Marks, 1995; Gallace & Spence, 2006; Palmer et al., 2013; Spence, 2020; Spence & Di Stefano, 2022; Velasco et al., 2015), and congruent pairings reliably influence performance in both behavioural and perceptual tasks (Mossbridge et al., 2011; Parise & Spence, 2008, 2009; Zeljko et al., 2019). The mechanisms underlying CMCs remain debated. Here, we use bottom-up to refer to stimulus-driven influences arising from sensory properties, perceptual coding biases, or implicit statistical regularities between stimulus dimensions (McEwan et al., 2024b; Parise et al., 2013; Parise, 2016; Spence, 2011), and top-down to refer to influences of task set, attention, expectations, relative coding, or strategic use of correspondence information (Brunetti et al., 2017; Chiou & Rich, 2012). This distinction is consistent with broader multisensory-attention frameworks in which stimulus-driven crossmodal interactions and top-down attentional control jointly shape perception, but we do not assume that these sources are mutually exclusive (Gilbert & Sigman, 2007; Parise, 2016; Spence & Deroy, 2013; Talsma et al., 2010).

Early research on CMCs relied on relatively simple paradigms such as speeded detection, discrimination, or classification tasks (Marks, 1987; Parise & Spence, 2012; Spence, 2011; Zeljko et al., 2019). These approaches established the robustness of congruency effects but provided limited insight into whether CMCs extend beyond low-level facilitation to influence higher-level perceptual processes. More recently, research has begun to explore whether CMCs can bias the resolution of perceptual ambiguity, where sensory input admits multiple plausible interpretations (McEwan et al., 2025; Zeljko et al., 2021). Ambiguous figures, such as the classic Rubin face–vase illusion (Rubin, 1915), offer a well-established means to investigate such effects: the same retinal input can be perceived either as two faces in profile or as a central vase, and perception alternates spontaneously between these interpretations (Long & Toppino, 2004; Sterzer et al., 2009).

Zeljko et al. (2021) tested whether the lightness/pitch correspondence could bias Rubin face–vase perception by pairing high- or low-pitched tones with black-and-white versions of the figure. They found that observers were more likely to perceive the interpretation congruent with the tone (white/high pitch, black/low pitch) relative to incongruent pairings. Crucially, the effect was observed only for longer stimulus durations and after prior exposure, suggesting that CMC-related biases emerge over time and may involve both bottom-up associations and top-down contextual learning. This finding demonstrates that CMCs can shape not only feature-level decisions but also the resolution of perceptual ambiguity at a representational level.

Building on this work, the present study examines whether CMCs can also bias reported dominance during binocular rivalry, a form of bistability arising when different images are presented to the two eyes, producing spontaneous perceptual alternations (Alais & Blake, 2005, 2014; Blake & Logothetis, 2002). Unlike figure–ground ambiguity in the Rubin face–vase illusion, binocular rivalry is thought to reflect competition across multiple levels of the visual hierarchy, including early visual processing. By testing whether CMCs bias perceptual outcomes under these conditions, we aim to assess whether their influence extends to a more fundamental and widely studied form of bistable perception.

When each eye is presented with a different image, visual perception does not settle on a fused combination but instead alternates between the two monocular inputs, a phenomenon known as binocular rivalry (Blake & Logothetis, 2002; Tong et al., 2006). During rivalry, conscious awareness switches every few seconds, granting dominance first to one image and then the other, despite constant sensory input. For simple small stimuli, rivalry tends to be “all-or-none”, with one image fully dominating while the other is strongly suppressed, whereas larger or more complex stimuli can give rise to mixed percepts with spatial mosaics of dominance and suppression (Alais & Blake, 2014; Logothetis et al., 1996). These perceptual fluctuations are stochastic and vary across individuals, but dominance durations typically fall in the range of one to several seconds (Alais & Blake, 2014; Miller et al., 2010; Nagamine et al., 2007).

The neural mechanisms of rivalry have been the subject of extensive debate. Early accounts emphasized low-level interocular competition, in which neurons tuned to each eye’s input inhibit one another (Blake, 1989; Tong et al., 2006). More recent evidence suggests rivalry is a multistage process, cascading from early visual competition through higher cortical levels (Blake & Wilson, 2011; Tong et al., 2006). At later stages, the suppressed image can be profoundly diminished from awareness, reflecting deep suppression mechanisms that extend well beyond attenuation of early sensory signals (Hense et al., 2019). Rivalry dynamics are sensitive to a wide range of factors, including local contrast, naturalistic contours, attention, expectation, and learning (Alais & Blake, 2014; Logothetis et al., 1996).

Crucially, rivalry can also be shaped by crossmodal inputs. Auditory or tactile signals can bias which of the competing images emerges into awareness or alter the timing of dominance switches (Alais et al., 2010; Alais & Blake, 2014; Conrad et al., 2010). In one striking demonstration, Plass et al. (2017) showed that congruent speech sounds biased rivalry toward ellipses resembling corresponding mouth shapes. Importantly, this effect involved speech-specific audiovisual congruency between heard speech sounds and visible mouth-shape dynamics, rather than a simple low-level mapping between arbitrary sounds and shapes. In more complex audio-visual interactions, Chen et al. (2011) found that auditory semantic context can bias visual dominance in binocular rivalry, while Hsiao et al. (2012) observed a similar semantic auditory effect on perceptual resolution of the wife/mother-in-law bistable image. Audiovisual congruence in these cases reflects learned semantic associations rather than untrained feature-based correspondences.

Kim and Kim (2019) provided further evidence that prior exposure is critical for crossmodal influences on rivalry. In their first experiment, rivalrous gratings flickering at 1 or 3 Hz were paired with tones of matching or mismatching frequency. Rivalry disambiguation, indexed by longer dominance durations and shorter piecemeal phases, emerged only after participants had explicitly learned the correct tone–grating pairings in a matching task. In a second experiment, arbitrary pairings between animal images and tones produced similar effects, again only after explicit training (Kim & Kim, 2019). These results highlight that crossmodal cues can bias rivalry, but primarily when associations are grounded in semantic knowledge or learned contingencies.

Taken together, current research shows that crossmodal inputs can influence rivalry dynamics, yet the evidence to date relies mainly on associations that are either semantically rich, such as speech-mouth shape pairings, or explicitly trained, such as tone-animal pairings. What remains largely unexplored is whether a pre-existing, untrained correspondence, such as the lightness/pitch CMC, can exert similar effects. Here, we use ‘implicit’ to distinguish pre-existing, untrained associations from those that are explicitly instructed, trained, or primed within an experimental paradigm. The lightness/pitch correspondence is considered implicit because participants were not taught a lightness–pitch rule, and any influence of pitch was inferred indirectly from rivalry dominance reports rather than from an explicit matching judgment. This use is consistent with prior CMC work using indirect measures of association, such as the Implicit Association Test (Anikin & Johansson, 2019; Parise & Spence, 2012). Importantly, however, we do not use ‘implicit’ to imply that the correspondence is necessarily unconscious, fully automatic, or independent of task demands. Demonstrating that such correspondences bias reported dominance would extend CMC effects to binocular rivalry.

Here, we investigated whether CMCs bias reported dominance during binocular rivalry by examining the effect of auditory pitch on the perceptual dominance of dichoptically presented black and white stimuli. Across a series of experiments, participants viewed rivalrous displays of a white circle to one eye and a black circle to the other, accompanied by a simultaneous tone that was either high or low in pitch, and reported their dominant percept. We measured dominance reports under both intermittent (static) and continuous (dynamic) presentation regimes and assessed whether congruent pairings (white/high pitch, black/low pitch) increased the likelihood of a congruent dominance report relative to incongruent pairings.

For the intermittent presentation, rivalrous displays accompanied by a high or low tone were presented for a short duration and then a single dominant percept response was made. For continuous presentation, rivalrous displays were presented for a longer duration while the tone regularly alternated between high and low pitch. For the continuous presentation, participants continuously reported their dominant percept as it alternated. This design allowed us to test whether CMCs bias dominance reports immediately upon stimulus onset or emerge gradually across rivalry cycles.

By manipulating presentation duration and task demands across the three experiments, we aimed to test whether CMC effects emerge immediately or require prolonged exposure, and whether they act directly on visual lightness judgments or indirectly via secondary features. We hypothesized that reported rivalry dominance would be biased toward the CMC-congruent alternative. Such a pattern would indicate that pre-existing lightness/pitch correspondences can influence dominance reports during binocular rivalry, while allowing us to test whether this influence depends on task relevance and the temporal dynamics of rivalry.

## Methods

### Participants

One hundred and seventy-nine undergraduate students (133 female, average age 21.5 years) from the University of Queensland participated in the study in return for course credit or a $20 AUD shopping voucher. We aimed for approximately 50 participants in each experimental condition to detect a small to medium effect size using one-sided *t*-test comparisons with α = 0.05 and 80% power (G*Power v3.1.9.4; Faul et al., 2007). The participants were allocated across three experiments as follows: 50 participants in Experiment 1, 79 participants in Experiment 2, and 50 participants in Experiment 3. Participant allocations to experimental conditions are described below.

All participants reported normal hearing and normal or corrected-to-normal vision, and all were naïve as to the purpose of the experiment. All experiments were cleared in accordance with the ethical review processes of the University of Queensland and within the guidelines of the National Statement on Ethical Conduct in Human Research.

### Apparatus and stimuli

Stimuli were generated on a Mac Pro computer using MATLAB (R2017a; MathWorks, Natick, MA, USA) and the Psychophysics Toolbox extensions (V3.0.11; Brainard, 1997; Kleiner et al., 2007) and presented on two 24-in. Macintosh cinema displays. The displays were viewed via a mirror stereoscope with two front-silvered mirrors oriented at ± 45° to the median plane of the head projecting each display to a different eye. Rectangular apertures were placed between the mirrors and their display screens to restrict the participants’ field of view to the display. Participants’ heads were restrained in a chin and forehead rest, which was adjusted for the participants to ensure their pupils were at the same height as the centre of the displays. Auditory stimuli were presented binaurally via headphones and responses were registered using the left and right arrow keys of a standard keyboard.

**Fig. 1:**
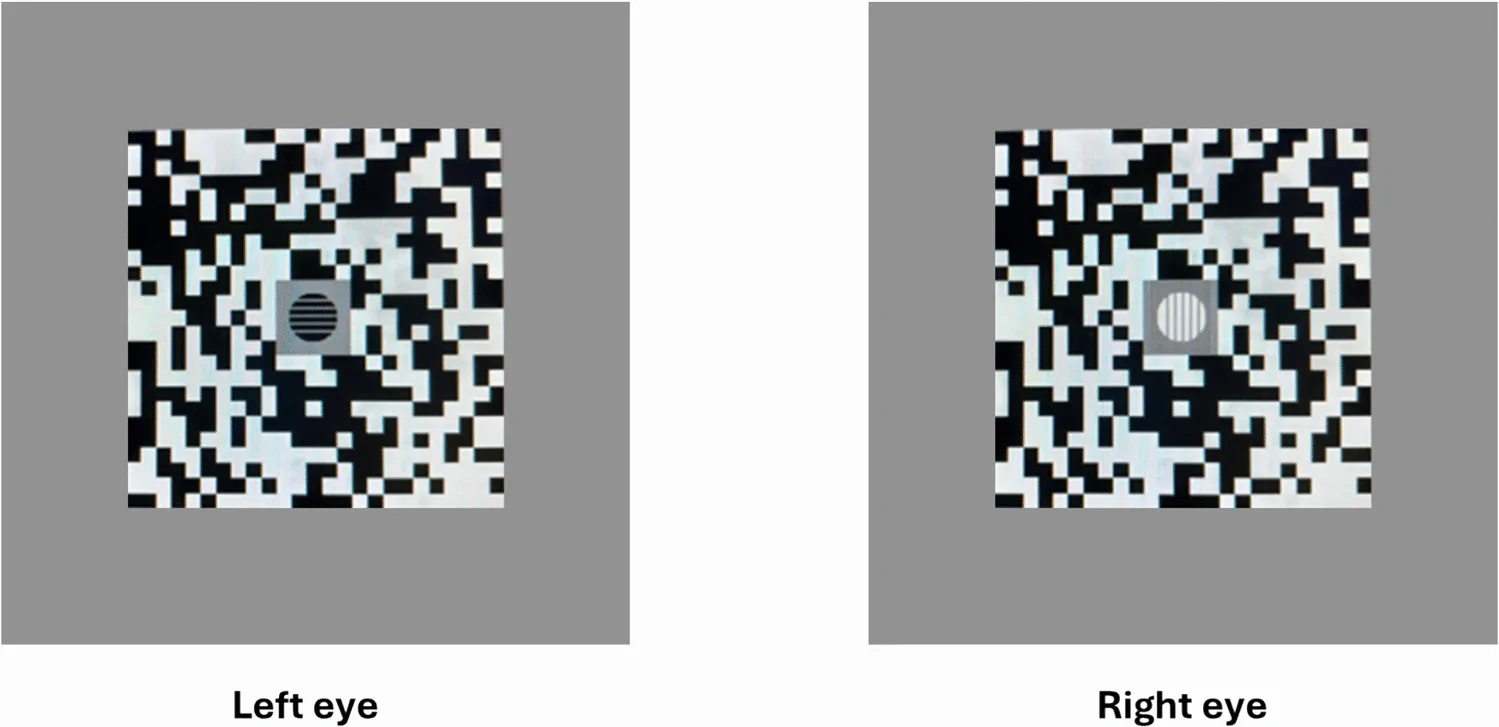
Example of a visual stimulus combination with black/horizontal presented to the left eye and white/vertical presented to the right eye

The visual stimuli were filled circles (black: RGB 0, 0, 0 or white: RGB: 255, 255, 255) one degree of visual angle in size and presented at the centre of each screen on a square mid-grey background (1.7 degrees). The filled circles had five equally spaced mid-grey lines (2 pixels width) positioned either horizontally or vertically such that there were four different visual stimuli (black/horizontal, black/vertical, white/horizontal, and white/vertical). Surrounding the mid-grey background was a binocular fusion lock consisting of a square black and white random noise pattern of 25 by 25 elements and subtending 8.4 degrees. The left- and right-side monitors always displayed a single visual stimulus of opposing colour and line orientation (see Fig. 1). The auditory stimulus was either a 300-Hz simple tone or a 1,800-Hz simple tone, and these were classified as ‘low’ and ‘high’, respectively, based on relative comparison. The tone was presented at a clear but self-reported comfortable volume level.

### Design

Three separate experiments were conducted with the stimulus presentation style, presentation duration, and task varying across ten experimental conditions. Each experimental condition was entirely within subjects, and all participants completed at least one intermittent condition and most also completed a continuous condition. While many participants completed more than one intermittent condition, no participants completed more than a single continuous condition, and no statistical comparisons were made between intermittent conditions.

There were two different stimulus presentation styles: intermittent and continuous. In the intermittent presentation conditions, each trial consisted of the brief simultaneous presentation of a rivalrous visual stimulus and one of the auditory stimuli (either the low or high tone), and participants reported their dominant visual percept after the fact. In the continuous presentation conditions, each trial consisted of the continuous presentation of a rivalrous visual stimulus and an auditory stimulus over an extended period and participants reported their dominant percept continuously as they were experiencing it. While the rivalrous visual stimulus remained fixed throughout each continuous presentation trial, the auditory stimulus varied alternately between the low and the high tones.

Experiment 1 consisted of two intermittent presentation conditions (presentation duration: 0.1 s and 1.2 s) and one continuous presentation condition (stimulus presentation: 70 s; tone alternation: 2 s). The task in Experiment 1 was to report lightness dominance (black or white). The 50 participants allocated to Experiment 1 each participated in each of the three experimental conditions. Experiment 2 consisted of three intermittent presentation conditions (presentation duration: 3 s, 5 s, and 8 s) and one continuous presentation condition (stimulus presentation: 70 s; tone alternation: 5 s). The task in Experiment 2 was to report lightness dominance (black or white). The 79 participants allocated to Experiment 2 completed partially overlapping subsets of the intermittent and continuous conditions (3 s: 57 participants; 5 s: 49 participants; 8 s: 57 participants; continuous: 51 participants). Experiment 3 consisted of two intermittent presentation conditions (presentation duration: 5 s and 8 s) and one continuous presentation condition (stimulus presentation: 70 s; tone alternation: 5 s). The task in Experiment 3 was to report orientation dominance (vertical or horizontal). The 50 participants allocated to Experiment 3 each participated in each of the three experimental conditions.

### Procedure

The basic operation of the mirror stereoscope and binocular rivalry was first described to each participant. They were told that two different circles would be presented, one to each eye, and that they should expect to experience an alternation between the two, and that there would be a simultaneous auditory tone. Participants were told that the visual alternations would likely be random, of varying length, and sometimes may be partial. The task in all experiments was to report which visual stimulus was perceptually dominant with the criteria for dominance described as the image that ‘appeared to be winning the competition’ for perception. While the same stimuli were presented in all experiments, for Experiments 1 and 2, they were described as black or white filled circles, and for Experiment 3 they were described as circles with vertical or horizontal stripes. Participants were instructed to make their best judgement without thinking about it too much.

Participants completed the blocks to which they were assigned within a single experimental session. Intermittent blocks were run first in counterbalanced order, and the continuous block, where completed, was run last. Each full participant session took approximately 40 min.

**Fig. 2:**
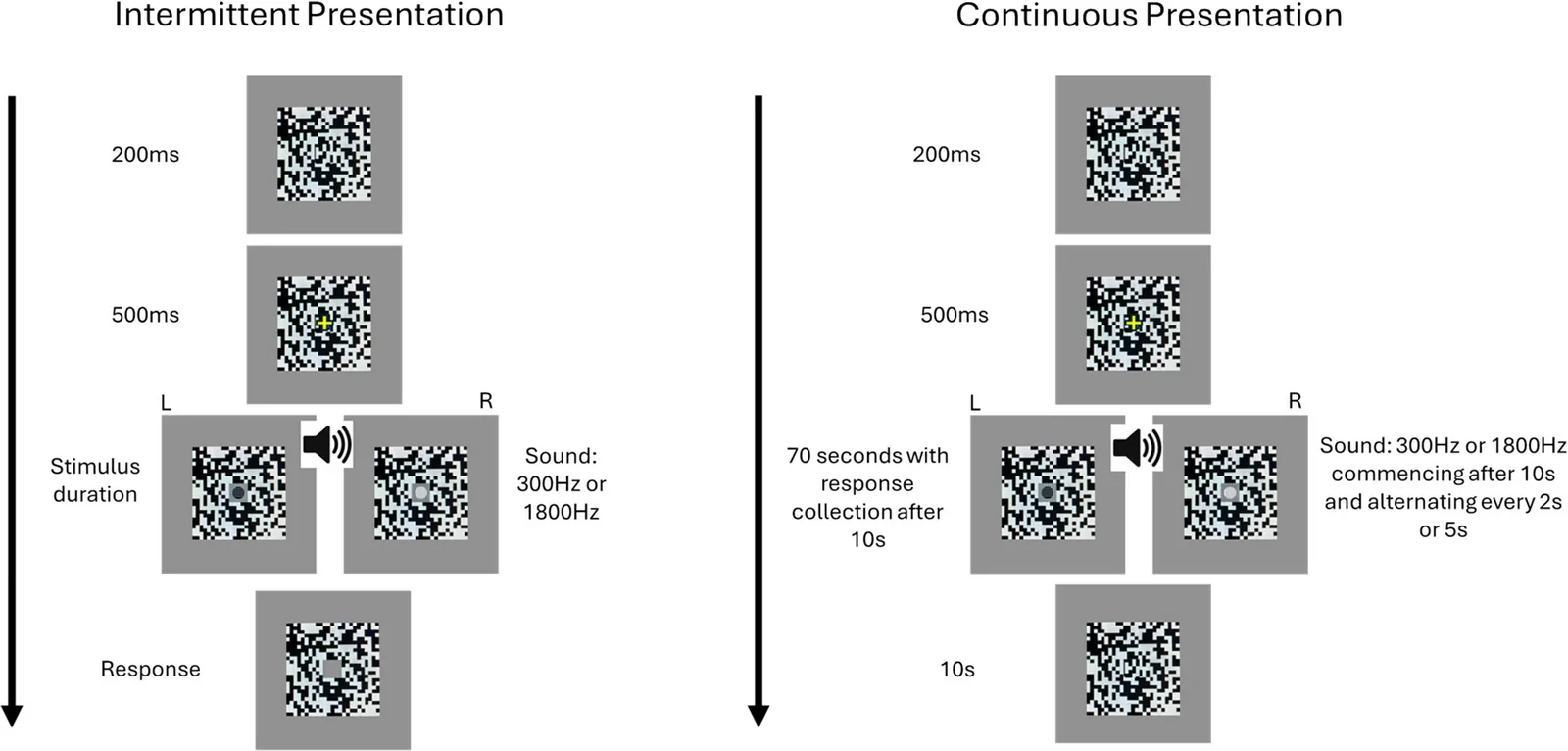
Trial sequence in each experiment. Example trial sequence for the intermittent presentation blocks (**left panel**) and the continuous presentation blocks (**right panel**) in each experiment

Each trial of an intermittent condition consisted of the following sequence of events. The fusion lock random noise pattern was presented to each eye for the entire trial. For the first 200 ms only the fusion lock pattern was presented, after which a yellow fixation cross was added. After 500 ms, the visual and auditory stimuli were presented for the stimulus duration which depended on the condition (0.1 s, 1.2 s, 3 s, 5 s, or 8 s), after which the visual stimuli were removed, leaving only the grey square in the centre of the fusion lock pattern. This remained until a response was made and only responses made after stimulus offset were recorded (Fig. 2). The left arrow was used for black and vertical responses and the right arrow for white and horizontal responses. There were 80 trials in total for each intermittent block with equal randomised presentations of each visual stimulus combination and each auditory stimulus.

Each trial of the continuous blocks consisted of the following sequence of events. The fusion lock pattern was presented to each eye for the entire trial. For the first 200 ms only the fusion lock pattern was presented, after which a yellow fixation cross was added. After 500 ms, the visual stimuli were presented for 70 s. Participants were instructed to commence continuous responses (left arrow for black and vertical and right arrow for white and horizontal) immediately upon the presentation of the visual stimuli, but responses were not recorded for the first 10 s to allow for rivalry to stabilise. After 10 s the sound onset commencing with either the low or the high tone (counter-balanced and alternating throughout the continuous presentation) and response sampling commenced at 50 Hz. Response sampling was halted after 60 s, and the visual stimuli and sound offset, leaving only the fusion lock pattern for 10 s (Fig. 2). There were eight trials in total for each continuous block with equal randomised presentation of each visual stimulus combination.

### Analyses

For each stimulus duration block of the intermittently presented stimuli in Experiments 1 and 2, participant trial-by-trial responses of black or white were transformed into congruent or incongruent responses (mapped 1 and 0, respectively), depending on the pitch of the sound during that trial. For Experiment 3, responses of vertical or horizontal were first mapped to the corresponding circle lightness (black or white) and then transformed into congruent or incongruent responses in the same way. Because high- and low-pitch tones occurred equally often, and because each tone had one congruent and one incongruent possible report, the null expectation was 50% congruent responses. This coding is equivalent to a conventional congruent-minus-incongruent difference score. We report the proportion of congruent responses because it provides an intuitive chance-level benchmark for the binary rivalry-report task. Because the theoretical prediction was directional, the primary tests of the intermittent rivalry blocks were one-sided. Specifically, prior work on lightness/pitch correspondences predicts that relatively high-pitched tones should be associated with lighter visual percepts and relatively low-pitched tones with darker visual percepts. The predicted effect was therefore a proportion of congruent reports greater than 50%. A proportion below 50% would not support the hypothesised correspondence effect but would instead indicate an unexpected reversal. One-sided tests are appropriate when the alternative hypothesis is directional and the opposite direction would not be interpreted as evidence for the same theoretical claim (Abelson, 1995; Ruxton & Neuhäuser, 2010).

For the continuous presentation blocks, participant continuous responses of black or white were transformed into congruent or incongruent responses (mapped 1 and 0, respectively) depending on the pitch of the sound during response (for Experiment 3, responses of vertical or horizontal were first mapped to black or white). Participant responses were then averaged to determine the percentage of congruent responses, and these were used as the basis for statistical comparison. Statistical tests were single-sample right-side *t*-tests of congruent responses against 50% based on the hypothesis that a congruency effect would manifest as congruent responses of greater than 50%.

In addition to a simple congruency effect, we also examined responses in the continuous blocks in terms of their congruency with the sound at various offsets from the response (with the sound leading the response). We re-transformed participant black or white responses as congruent or incongruent depending on the pitch of the sound at times before the response was made and then for each offset time. Specifically, we examined response offset times ranging from 0.1 s after the sound to 2 s after the sound (Experiment 1) or 5 s after the sound (Experiments 2 and 3) in steps of 0.1 s. For each response offset time, we determined the average congruent responses for the entire block for each participant, then determined the group mean congruent responses and compared these to 50% using single-sample right-side *t*-tests.

Finally, we examined the dominance duration of the rivalrous images in the continuous blocks by determining the duration of each consecutive black or white (or vertical or horizontal) response for each participant, then averaging these to determine the group means.

## Results

### Experiment 1: Short duration/lightness dominance

Experiment 1 comprised two blocks of intermittent presentation trials with stimulus durations of 0.1 and 1.2 s and a single block of 60-s continuous presentation trials with the sound alternating between high and low pitch every 2 s. Participants reported whether the black- or white-filled circle was dominant.

#### Intermittent presentation

**Fig. 3:**
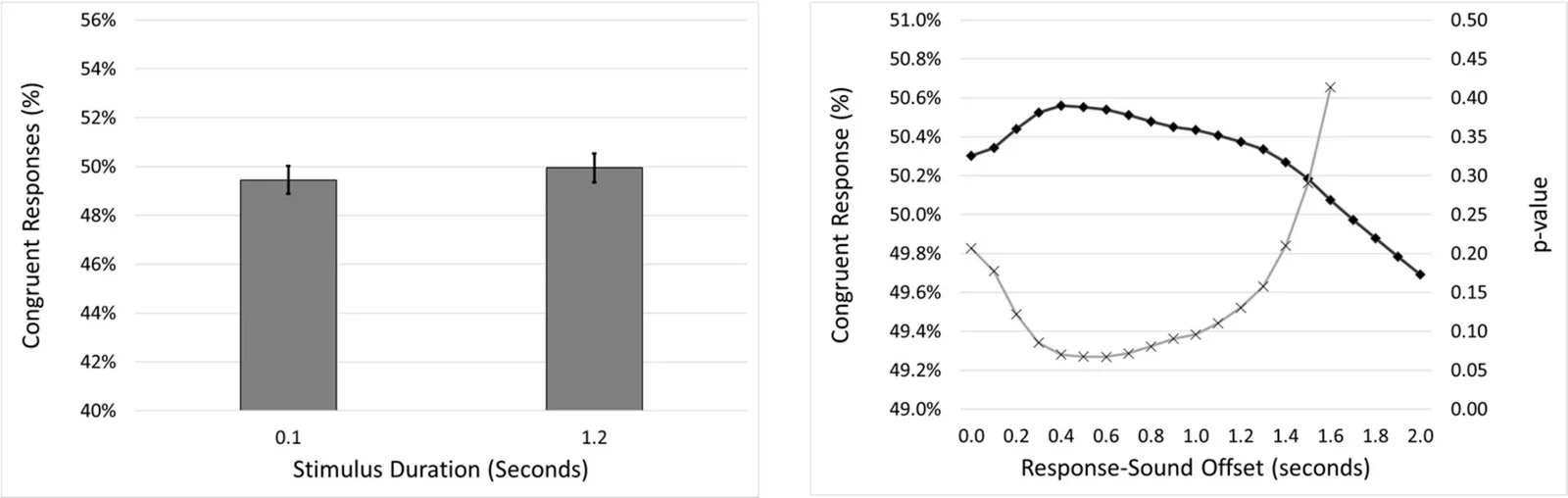
**Left:** Group mean percentage of congruent responses to intermittent stimulus presentation for each stimulus duration in Experiment 1 (error bars are the standard error of the mean). **Right:** Group mean percentage of congruent responses (black lines) and corresponding *p*-value for comparisons against 50% for each sound-response offset time for the continuous stimulus presentation in Experiment 1

The data from four participants were excluded from the 0.1-s analysis and five participants from the 1.2-s analysis due to either response bias (responding only black or white for all trials) or eye dominance (responding only to the left or right image on all trials). We found no evidence for congruency effects for either the 0.1-s (*M* = 49.4%, *SE* = 0.6%, *t*(45) = −0.97, *p* = 0.830) or the 1.2-s (*M* = 50.0%, *SE* = 0.6%, *t*(44) = −0.09, *p* = 0.536) stimulus duration conditions (see Fig. 3).

#### Continuous presentation

The group mean percent of congruent responses was 50.3%, which did not differ significantly from 50% (*t*(49) = 0.82, *p* = 0.207). For temporally offset responses, the group mean of congruent responses increased smoothly to a maximum value then decreased as the offset duration increased while the corresponding *p*-values similarly decreased to a minimum value then increased (see Fig. 3). The maximum congruency effect was observed when the response followed the sound by 0.4 s (*M* = 50.6%, *SE* = 0.3%) but it did not reach significance (*t*(49) = 1.50, *p* = 0.070).

The average dominance response duration was 2.57 s (*SD* = 0.89 s; one participant’s average dominance response duration was over 5 *SD*s from the mean and so was excluded).

### Experiment 2: Long duration/lightness dominance

Experiment 2 comprised three blocks of intermittent presentation trials with stimulus durations of 3, 5, and 8 s and a single block of 60-s continuous presentation trials with the sound alternating between high and low pitch every 5 s. Participants reported whether the black or white image was dominant.

#### Intermittent presentation

**Fig. 4:**
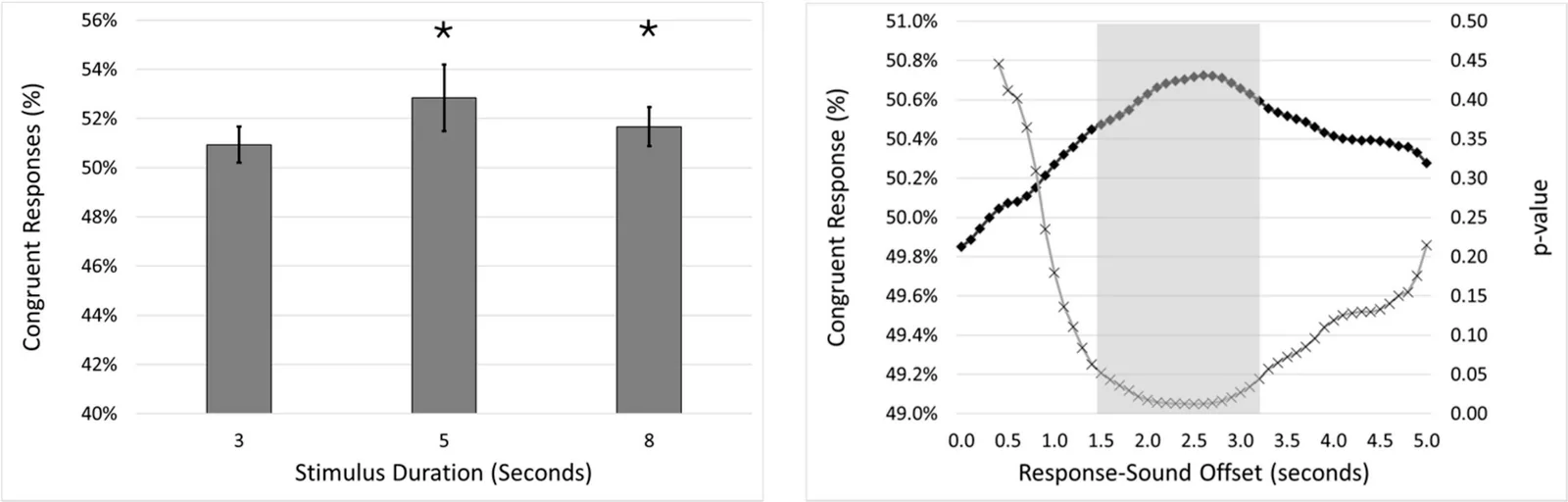
**Left:** Group mean percentage of congruent responses to intermittent stimulus presentation for each stimulus duration in Experiment 2. Error bars are the standard error of the mean. Asterisks indicate raw planned one-sided *p* <.05. **Right:** Group mean percentage of congruent responses and corresponding pointwise *p*-values for one-sided comparisons against 50% at each sound-response offset time in the continuous presentation condition. The grey-shaded area indicates offset times for which the pointwise comparison was significant at *p* <.05, uncorrected

The data from one participant were excluded from the 3-s analysis and one participant from the 5-s analysis due to either response bias (responding only black or white for all trials) or eye dominance (responding only to the left or right image on all trials). Planned one-sided tests indicated congruency effects in the 5-s stimulus duration condition (*M* = 52.8%, *SE* = 1.4%, *t*(47) = 2.10, raw *p* =.021, *d* = 0.30) and in the 8-s stimulus duration condition (*M* = 51.7%, *SE* = 0.8%, *t*(56) = 2.11, raw *p* =.020, *d* = 0.28). There was no congruency effect in the 3-s stimulus duration condition (*M* = 50.9%, *SE* = 0.7%, *t*(55) = 1.28, raw *p* =.102) (see Fig. 4). These planned tests were not adjusted for multiple comparisons; under a conservative Holm correction across the three Experiment 2 intermittent durations, neither the 5-s nor the 8-s effect survived correction.

#### Continuous presentation

Data from two participants were excluded because their sampled responses represented less than 50% of the total presentation duration. The group mean percent of congruent responses was 49.9%, which did not differ significantly from 50% (*t*(48) = −0.45, *p* = 0.673). For temporally offset responses, the group mean of congruent responses increased smoothly to a maximum value then decreased as the offset duration increased while the corresponding *p*-values similarly decreased to a minimum value then increased (see Fig. 4). The congruency effect was statistically significant for response offsets between 1.6 s (*M* = 50.5%, *SE* = 0.3%, *t*(48) = 1.75, *p* = 0.043, *d* = 0.25) and 3.2 s (*M* = 50.6%, *SE* = 0.3%, *t*(48) = 1.74, *p* = 0.044, *d* = 0.25). The maximum congruency effect was observed when the response followed the sound by 2.6 s (*M* = 50.7%, *SE* = 0.3%), which was statistically significant with a small to medium effect size (*t*(48) = 2.32, *p* = 0.012, *d* = 0.33).

The average dominance response duration was 2.51 s (*SD* = 1.00 s; a further seven participants’ average dominance response durations were over 3 *SD*s from the mean, and so were excluded). We lastly compared the average dominance response durations between Experiment 1 (2-s alternating sound) and Experiment 2 (5-s alternating sound) using an independent-samples *t*-test, and found that they were not significantly different (*t*(90) = 0.312, *p* = 0.756).

### Experiment 3: Long duration/orientation dominance

Experiment 3 comprised two blocks of intermittent presentation trials with stimulus durations of 5 and 8 s and a single block of 60-s continuous presentation trials with the sound alternating between high and low pitch every 5 s. Participants reported whether the vertical or horizontal image was dominant.

#### Intermittent presentation

**Fig. 5:**
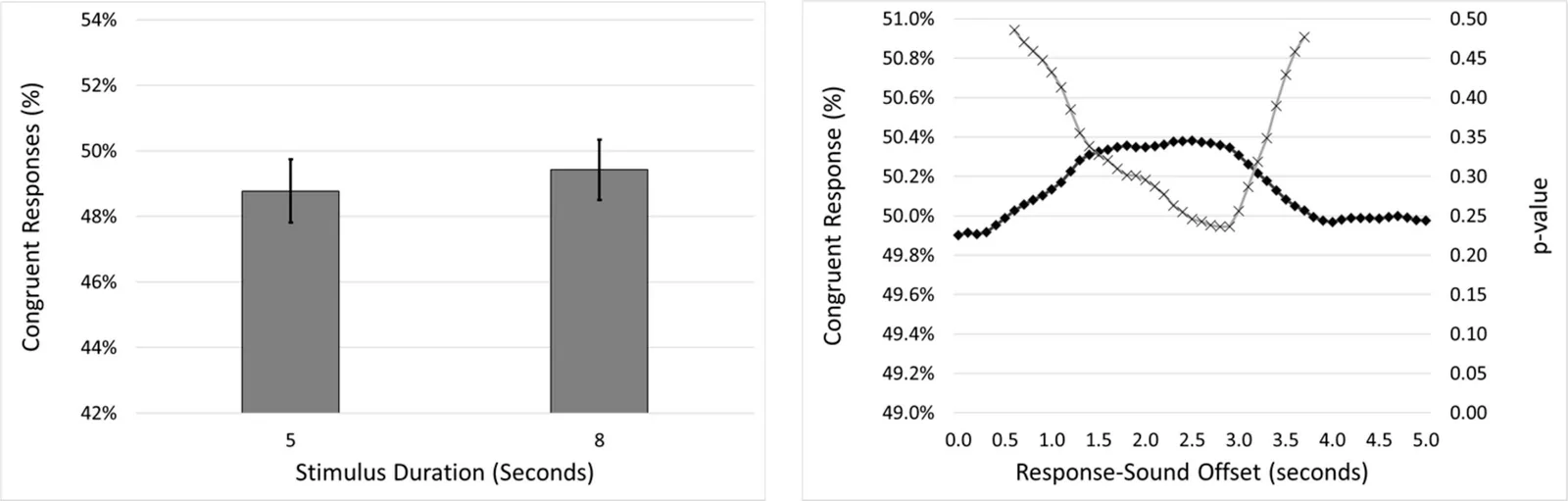
**Left:** Group mean percentage of congruent responses to intermittent stimulus presentation for each stimulus duration in Experiment 3 (error bars are the standard error of the mean). **Right:** Group mean percentage of congruent responses (black lines) and corresponding *p*-values for comparisons against 50% for each sound-response offset time for the continuous stimulus presentation in Experiment 3

The data from three participants were excluded from the 5-s analysis and from two participants from the 8-s analysis due to either response bias (responding only vertical or horizontal for all trials) or eye dominance (responding only to the left or right image on all trials). We found no evidence for congruency effects for either the 5-s (*M* = 48.8%, *SE* = 1.0%, *t*(46) = −1.27, *p* = 0.895) or the 8-s (*M* = 49.4%, *SE* = 0.9%, *t*(47) = −0.63, *p* = 0.733) stimulus-duration conditions (see Fig. 5).

#### Continuous presentation

Data from 12 participants were excluded because their sampled responses represented less than 50% of the total presentation duration. The group mean percent of congruent responses was 49.9%, which did not differ significantly from 50% (*t*(37) = −0.14, *p* = 0.559). For temporally offset responses, the group mean of congruent responses increased smoothly to a maximum value then decreased as the offset duration increased while the corresponding *p*-values similarly decreased to a minimum value then increased (see Fig. 5). The maximum congruency effect was observed when the response followed the sound by 2.5 s (*M* = 50.4%, *SE* = 0.5%), but it was not statistically significant (*t*(37) = 0.69, *p* = 0.246).

The average dominance response duration was 3.52 s (*SD* = 2.15 s; a further participant’s average dominance response duration was over 4 *SD*s from the mean and so was excluded). We lastly compared the average dominance response duration in Experiment 3 (5-s alternating sound) with those in Experiment 1 (2-s alternating sound) and Experiment 2 (5-s alternating sound) using independent-samples *t*-tests, and found that it was significantly greater in both cases (Experiment 1: *t*(78) = 2.71, *p* = 0.008, *d* = 0.61; Experiment 2: *t*(84) = 2.75, *p* = 0.007, *d* = 0.60).

## Discussion

The present study examined whether crossmodal correspondences (CMCs) bias reported perceptual dominance during binocular rivalry. Specifically, we tested whether auditory pitch biases reports of dominance between dichoptically presented black and white stimuli, consistent with the lightness/pitch correspondence that links relatively high pitch with lighter stimuli and relatively low pitch with darker stimuli. Based on prior evidence that CMCs can modulate the resolution of higher-level perceptual ambiguity, such as the Rubin face–vase illusion (Zeljko et al., 2021), we hypothesised that reported rivalry dominance would be biased in favour of the CMC-congruent visual alternative. By testing this within a rivalry paradigm, in which competing monocular inputs vie for awareness, we aimed to determine whether pre-existing pitch/lightness correspondences extend beyond response facilitation and figure–ground ambiguity to influence reports of perceptual dominance under conditions of direct visual competition.

Across the three experiments, the results provided partial support for this hypothesis, but critically the effects depended on both task relevance and stimulus timing. In the direct task (Experiments 1 and 2), where participants reported whether the dominant percept was black or white, congruency effects emerged under both intermittent and continuous presentation, but only at intermediate time scales. For intermittent presentation, a significant congruency effect was observed at the longer durations (5 s and 8 s), with no effect at shorter (0.1–3 s) durations. In the continuous condition, congruency effects were again specific to 5-s but not 2-s sound alternations, with significant biases toward congruent dominance reports occurring when responses followed tone changes by 1.6–3.2 s and peaking at about 2.6 s with a small to medium effect size. These were planned directional tests for congruency effects and have not been adjusted for multiple comparisons. We interpret these findings cautiously since, under a conservative adjustment (Bonferroni-Holm) the longer duration congruency effects fail to reach significance; however, we note that the pattern of results (longer vs. shorter duration) and the significant effect in the longer duration continuous condition both support our conclusion.

By contrast, in the indirect task (Experiment 3), where participants judged vertical versus horizontal dominance while lightness was irrelevant, no congruency effects were observed under either intermittent or continuous presentation, although the null effect in the continuous condition should be interpreted cautiously because 12 of 50 participants were excluded, leaving a final sample of 38 and reducing sensitivity to smaller effects. Finally, analysis of dominance response durations in the continuous condition showed that dominance epochs averaged about 2.5 s in the direct task (both 2-s and 5-s sound alternations) but were significantly longer at about 3.5 s in the indirect task during 5-s sound alternation.

The dependence of congruency effects on task relevance in the present study parallels and extends earlier findings. In our Rubin face–vase study, CMCs biased reports of figure–ground interpretation even though lightness was not the explicit response dimension (Zeljko et al., 2021), suggesting that under some conditions correspondences can influence the resolution of perceptual ambiguity indirectly. By contrast, our signal-detection/reaction-time work using simple pitch–lightness pairings found effects only when participants judged the CMC-relevant feature directly, with no influence when they judged an orthogonal feature (Zeljko et al., 2019), a pattern that aligns with the present results. Previously, Evans and Treisman (2010) reported congruency effects in both direct and indirect tasks for pitch–size and pitch–position correspondences, highlighting that task-dependence varies across correspondence domains and paradigms. This variability is consistent with arguments that audiovisual correspondences should not be treated as obligatorily automatic, and that their expression can depend on instructions, attentional set, and task demands (Getz & Kubovy, 2018; Spence & Deroy, 2013). Taken together, these comparisons suggest that CMCs are not uniformly automatic. In the present study, the lightness/pitch correspondence biased performance only when lightness was task-relevant, indicating that its effect was strongly gated by whether the relevant feature was attended.

Beyond task relevance, these results highlight a striking temporal dependency. Congruency effects were observed only at intermediate timescales, converging across intermittent and continuous conditions. In the continuous task, the peak effect occurred when perceptual reports followed tone alternations by ~2.5 s, closely matching the average dominance duration. This pattern suggests that correspondences do not produce an immediate and sustained change in reported dominance but instead exert their strongest influence at a delay consistent with the timing of rivalry transitions. In the intermittent task, congruency effects appeared only at longer (5 s and 8 s) exposures and shorter presentations (0.1–3 s) likely ended before a congruency bias could manifest. This interpretation is consistent with established rivalry dynamics: alternations typically occur every 2–3 s (Alais & Blake, 2014; Miller et al., 2010), and external signals are more likely to shape perception during transition phases than during periods of stable dominance (Blake & Wilson, 2011). Event-related potential (ERP) studies also support this timing account, showing both early (~100–130 ms) and later (approximately 400–600 ms) components associated with perceptual reversals (Kornmeier & Bach, 2004; Pitts & Britz, 2011). Our results fit within this framework, suggesting that CMC-related biases in reported dominance are most evident during windows of instability rather than continuously throughout dominance phases.

Because rivalry dominance was measured by report, the present findings should be interpreted as effects on reported dominance rather than as decisive evidence for a purely perceptual change. The congruency coding minimises non-specific response biases: congruent responses included both visual alternatives, namely white reports during high-pitch tones and black reports during low-pitch tones, whereas incongruent responses included white reports during low-pitch tones and black reports during high-pitch tones. Thus, an overall tendency to report one colour, favour one response key, or favour one eye/side of presentation would not by itself shift congruent responding above chance, provided that the bias was independent of tone. However, the design cannot fully exclude a tone-contingent decision or report bias, particularly in the direct lightness-report tasks, in which a high tone could increase the likelihood of a white response, or a low tone could increase the likelihood of a black response. Several aspects of the data argue against a simple, non-specific response-bias account, including the duration dependence of the effect, its temporal alignment with rivalry dominance durations, and its absence when lightness was task irrelevant in Experiment 3. Nevertheless, future work using objective measures of dominance, response-mapping controls, or signal-detection approaches would be needed to separate perceptual modulation from report-level influences more conclusively.

These temporal dynamics distinguish pre-existing pitch/lightness correspondences from other forms of crossmodal influence on rivalry. Plass et al. (2017) demonstrated that congruent speech sounds biased rivalry toward ellipses resembling mouth shapes, an effect involving familiar audiovisual speech correspondences between heard phonemes and visible mouth-shape dynamics. Kim and Kim (2019) showed that both sensory (tone–grating) and arbitrary (tone–animal) pairings could bias rivalry, but only after explicit training, highlighting the role of prior learning. Relatedly, Klapetek et al. (2012) found that pitch–brightness congruency did not modulate the pip-and-pop visual-search effect when congruent and incongruent trials were randomly intermixed; a congruency effect emerged only when cue congruency was blocked, and participants were informed about the pitch–brightness mapping. Thus, even for a putatively natural pitch–brightness correspondence, expression of the effect can depend on explicit task structure or top-down facilitation. Similarly, tactile–visual interactions have been shown to bias rivalry, but often in an additive manner or under specific timing constraints (Hense et al., 2019; Lunghi & Morrone, 2013; Lunghi et al., 2014). Compared with these relatively robust, semantic, or explicitly supported effects, the present results indicate that pre-existing, untrained, and non-predictive crossmodal correspondences can bias reported rivalry dominance, but in a weaker and more temporally constrained manner.

Mechanistically, these findings are most consistent with a constrained hybrid account of CMC influence, while leaving open the origin of the lightness/pitch correspondence itself. Prior work indicates that CMC effects can include sensory/perceptual components as well as task- and context-dependent components. For example, signal-detection studies have shown congruency-related changes in sensitivity without corresponding criterion shifts, supporting a sensory contribution to at least some pitch-based CMCs (Mossbridge et al., 2011; Zeljko et al., 2019). At the same time, the relative nature of many pitch-based correspondences (Spence, 2019) and their sensitivity to task relevance indicate that top-down factors such as context, attention, and response goals can modulate whether a correspondence is expressed. The present results fit this mixed picture. The direct-task effects are consistent with the possibility that pitch/lightness congruency biases perceptual selection during rivalry, but because the measure was report-based, they may also include a tone-contingent decisional component. The absence of effects in the indirect task indicates that the correspondence is not automatically applied in all contexts; its influence depends on whether the relevant visual feature is attended and behaviourally relevant.

A Bayesian interpretation can accommodate this pattern without requiring a purely perceptual account. Congruent correspondences may increase the effective weight or prior probability of a given perceptual/report outcome, but this influence is expressed primarily when the visual system is unstable and multiple interpretations are plausible. Under this account, attention determines which stimulus dimensions are weighted, while rivalry dynamics determine when those weights can most effectively bias reported dominance.

In conclusion, the present study provides evidence that a pre-existing, untrained pitch/lightness correspondence can bias reported dominance during binocular rivalry. These effects were not automatic or continuous but depended on task relevance and were temporally aligned with natural rivalry dynamics. The findings therefore suggest that CMCs can function as subtle multisensory cues that bias reported perceptual outcomes during windows of instability. Although the present design cannot conclusively separate perceptual modulation from tone-contingent report bias, the timing profile, task dependence, and convergence with prior CMC work support the view that pitch/lightness correspondence influences more than simple response facilitation. By extending CMC effects beyond speeded classification tasks and high-level figure–ground ambiguity into binocular rivalry, the present findings highlight the broader role of crossmodal correspondences in shaping perceptual reports under conditions of direct sensory conflict.

## Data Availability

The data for this study are publicly available on the Open Science Framework (OSF) at: https://osf.io/u3ycm/overview?view_only=9fc10ddf4d6c4af8a7d70f2e06c2aef6. The study was not preregistered.

## Code availability

The experimental code used in this study is available from the corresponding author upon reasonable request.
